# Mental health and COVID-19: is the virus racist?

**DOI:** 10.1192/bjp.2020.93

**Published:** 2020-05-05

**Authors:** Anuj Kapilashrami, Kamaldeep Bhui

**Affiliations:** 1 Centre for Global Public Health, Institute of Population Health Sciences, Barts and The London School of Medicine and Dentistry, Queen Mary University of London, UK; 2 Centre for Psychiatry, Wolfson Institute of Preventive Medicine, Barts and The London School of Medicine and Dentistry, Queen Mary University of London; and East London NHS Foundation Trust, UK

**Keywords:** COVID-19, mental illness, inequalities, transcultural psychiatry, public health

## Abstract

COVID-19 has changed our lives and it appears to be especially harmful for some groups more than others. Black and Asian ethnic minorities are at particular risk and have reported greater mortality and intensive care needs. Mental illnesses are more common among Black and ethnic minorities, as are crisis care pathways including compulsory admission. This editorial sets out what might underlie these two phenomena, explaining how societal structures and disadvantage generate and can escalate inequalities in crises.

The COVID-19 coronavirus pandemic has dramatically changed the lives of people across the world, not only directly because of poor health with flu-like symptoms, hospital admission and death, but also indirectly by virtue of restrictions introduced to reduce infection. There are major consequences for businesses, employment, income, mobility, social contact and support, leisure and physical activity, and entrenched long-term health consequences due to isolation, bereavements and fears about infection; for those with existing conditions, there is a greater risk of deterioration and need for intensive care, and the very real possibility of the illness being fatal.^1,2^

As evidence on risks and burden emerges from different parts of the world, it is clear that COVID-19 is simultaneously an inequality amplifier and a stark reminder of the unequal world we inhabit.^3^ On the one hand, it exposes the deep-rooted social inequalities prevalent in society – differentially affecting the more vulnerable, for example, those in precarious employment, migrants and refugees, women in abusive relationships, those in receipt of care services and, in particular, those with existing mental health conditions. On the other, it calls for assessment of the disproportionate burden of the impact along different aspects of social location. Clarion calls to examine differences based on gender have more recently been joined by the need to examine race/ethnicity.

## How to explain racial/ethnic differences in COVID-19 mortality?

Recent media and official reports on deaths from COVID-19 in the USA and UK revealed excess mortality in Black and Asian minority ethnic communities and migrants as compared with the White American, British or European cohorts. The announcements of the deaths of doctors, nurses and social care workers on the front line with insufficient protective equipment, and similar data from the Intensive Care National Audit and Research Centre at Oxford, all suggest an excess of people from ethnic minorities needing ventilators and intensive care.^2^ Consequently, the media and public health gaze has shifted to the issue of racial/ethnic differences.

These startling findings caused a stir of speculations and demands for immediate investigation and remedial action. Indeed, Public Health England has launched an enquiry into COVID-19 deaths among Black and ethnic minorities and migrants. The explanations offered are varied and typify the fault-lines in health studies that have examined racial/ethnic differences in health outcomes. Three key explanations have dominated these studies:^4^ genetic and physiological vulnerabilities such as angiotensin-converting enzyme 2 (ACE2) receptor regulation and comorbidities to which minorities are more susceptible; cultural and behavioural factors that make for greater risks of infection and reveal the futility of universal public health messages; and sociological and structural conditions that are conducive to generating, sustaining and escalating inequalities. The first two dominate public health responses and strategies that tend to recommend better and more aggressive management of pre-existing conditions or focus on improving cultural competence and tackling linguistic barriers by, for example, the use of interpreters. Some responses have pathologised cultural and faith-based beliefs and practices, resulting in prejudice and, in some instances, racist rants.

The sociological and structural explanations emphasise socioeconomic disadvantage and overrepresentation of deaths and illness episodes in more deprived areas of greater unemployment and health risks, including poorer mental health and higher care needs. These areas experience greater demands on services; have crowded accommodation, with more resident migrants, refugees, homeless people and minorities; and have more people with lower levels of household income. These areas are also where more front-line workers are from minorities, and thus more exposed, but perhaps also where more are expected to continue to work as usual, even without protection – driven, presumably by their altruism and commitment to work and public service.

## Does ethnicity supersede socioeconomic disadvantage?

Yet the high rates of infection and death of healthcare workers from ethnic minorities raises questions about how ethnicity might supersede (and may act independently from) socioeconomic disadvantage. Interpretation of these data will have to account for greater concentration of ethnic minority populations in London and the West Midlands, where the UK outbreak is concentrated, as well as differentiate between healthcare workers and populations in general for their distinct experiences. For example, health literacy and cultural explanatory models for ‘pandemics’ may influence lay health risk behaviours and actions. Notwithstanding these differences, important questions need to be asked:

- Are specialties and services critical for the COVID-19 front-line response more likely to be staffed by people from ethnic minority groups and immigrants?
- To what extent and by what mechanisms does their greater propensity for chronic conditions translate into their susceptibility to coronavirus and poorer outcomes?
- Are they less likely to resist or raise concerns with regard to carrying out their duties with inadequate protection (e.g. personal protective equipment, PPE), longer work hours and lower pay, thereby putting them at increased risk of succumbing to the virus?

## Is there an NHS underclass?

Workforce race equality data have revealed inequalities in pay and career progression opportunities, and experiences of discrimination and bullying in the UK's National Health Service (NHS). Doctors and nurses and managers from ethnic minority groups are not earning as much as their counterparts or living in similar conditions and they carry allostatic loads and levels of chronic disease as a consequence of a combination of influences that seem to render them as vulnerable as those in lower socioeconomic positions and more vulnerable than those in higher socioeconomic positions in similar jobs and roles. The underlying implication is of an underclass signalled by ethnicity and embedded in social structures that work for the most wealthy and healthy and less so for those facing adversity, poverty and hardship, not only in that moment of crisis, but over the life course. Yet, these experiences cannot be homogenised for all minorities and migrant populations. Better data and an intersectional lens are needed to examine their susceptibility on the basis of gender, age, socioeconomic status, employment conditions and other stratifiers.^3^

## Inequalities in research fuel inequalities in solutions

Ethnic inequalities in the experience and outcomes of illnesses, especially mental illnesses, have a long research history of contested explanations and evidence that fails to capture the complexity of life-course adversity, combined with social structures and interactions with pathophysiologies. These cumulatively lead to poorer health through distress, discrimination and trauma, and marginalisation and disempowerment. Even among those in professions, work stress due to race inequality and discrimination still manifest to undermine the economy and healthy work environments. The lack of place-based and complex systems analyses is striking and may lie at the heart of failure to tackle these inequalities.

The same might be said of COVID-19. A greater rate of death due to pandemics and infectious disease among minorities is not a new finding. It is supported by evidence of past pandemics, albeit mostly from high-income counties.^5^ In lower-income countries, the data are poor, testing capacity is less and pandemic responses seek to save lives against the forces of extreme poverty and structures of disadvantage, including the inherent imbalance of power and resources between the global North and South. Many global South countries cannot afford a crisis, and key populations are unable to enforce distancing as rigidly as higher-income North countries because of population density, and crowded and precarious living and working conditions that mark informal economies. So, disparities will not be recorded or noted, but lost in the higher mortality rates.

## Crisis-driven legislative change furthers inequalities

More directly, a crisis response to COVID-19 has seen potentially harmful restrictions in socialisation and support that undermine mental health and compromise the care of people with established conditions. Legislation in the UK was hurried through parliament without public or patient consultation primarily to tackle anticipated workforce shortages by reducing the protections afforded by the Mental Health Act 1983. Ironically, this came after a review of the Act that called for greater protections to tackle ethnic and other inequalities in mental healthcare. These actions, although taken in crisis, reflect underlying structures and prioritisation of one form of crisis (COVID-19) over another (mental health emergency and loss of liberty), with potentially long-term and more catastrophic outcomes.

## When ‘normality’ is itself a state of crisis

So how has this parlous state of affairs arisen? The virus is not sentient or racist, but our social structures and reactions to crisis reflect values and power structures that continue to discriminate and determine poorer outcomes for some more so than others. What is surprising is it takes a crisis to highlight these inequalities and for us to take note, only to revert to the status quo once the crisis is over. While some wait for ‘normality’ to return, it is important we remind ourselves that, for many, ‘normal’ will continue to be a state of crisis. To avert the bigger global crisis that looms, at the least we need to gather better data on ethnicity and other aspects of social location. We then need to test preventive policies and systemic interventions to tackle clustered social disadvantage that is exploited in disease scenarios by nefarious viruses and reactive policies and practices that, however well intentioned, sustain and widen inequalities.
